# Drugs and Vaccines Hypersensitivity in Children with Mastocytosis

**DOI:** 10.3390/jcm11113153

**Published:** 2022-06-01

**Authors:** Francesca Mori, Giuseppe Crisafulli, Annamaria Bianchi, Paolo Bottau, Silvia Caimmi, Fabrizio Franceschini, Lucia Liotti, Claudia Paglialunga, Francesca Saretta, Carlo Caffarelli

**Affiliations:** 1 Allergologia, Dipartimento di Pediatria, Ospedale Anna Meyer, 50132 Firenze, Italy; francesca.mori@meyer.it; 2 UOC di Pediatria, Dipartimento Materno-Infantile, Università di Messina, 98168 Messina, Italy; crisafullig@unime.it; 3 UOC Pediatria, Ospedale San Camillo Forlanini, 00152 Roma, Italy; annamaria.bianchi9@gmail.com; 4 Pediatria e Neonatologia, Ospedale di Imola (BO), 40026 Imola, Italy; p.bottau@gmail.com; 5 UOC Pediatria Fondazione IRCSS Policlinico San Matteo, 27100 Pavia, Italy; silviamaria.caimmi@unipv.it; 6 UOC Pediatria, Azienda Ospedaliero-Universitaria “Ospedali Riuniti”, 60123 Ancona, Italy; allped@libero.it (F.F.); lucialiotti@libero.it (L.L.); 7 UOC di Pediatria, Azienda Ospedaliera-Universitaria Consorziale “Policlinico-Ospedale Pediatrico Giovanni XXIII”, 70120 Bari, Italy; clapag07@gmail.com; 8 SC Pediatria, Ospedale Latisana-Palmanova, Dipartimento Materno-Infantile Azienda Sanitaria Universitaria Friuli Centrale, 33100 Udine, Italy; francescasaretta@gmail.com; 9 Clinica Pediatrica, Dipartimento Medicina e Chirurgia, Università di Parma, 43126 Parma, Italy

**Keywords:** children, drug hypersensitivity, mastocytosis, vaccine hypersensitivity, beta-lactams, NSAID, perioperative allergy, antibiotics, biologics

## Abstract

Mastocytosis, a heterogeneous mastcell disease, include three different entities: cutaneous mastocytosis, systemic mastocytosis (SM) and mast-cell sarcoma. Tryptase levels can differentiate cutaneous mastocytosis from SM. In mastocytosis, quick onset drug hypersensitivity reactions (DHRs) that are facilitated by mastcell mediators, are investigated in adults. Due to the limited number of children with mastcell disease and increased serum tryptase levels, the role of drugs in this age group is less studied. In this review, we critically assessed relevant papers related with immediate DHRs in children with mastocytosis and discuss practical issues of the management. In childhood mastocytosis, anaphylaxis is frequently idiopathic, and elevated level of basal tryptase, and high burden of disease may increase the risk. Among drugs, antibiotics, NSAIDs and opioids can potentially induce anaphylaxis, anyway avoidance should be recommended only in case of previous reactions. Moreover, vaccinations are not contraindicated in patients with mastocytosis. The risk of severe systemic reactions after drugs intake seems to be extremely low and in general lower in children than in adults. Anyway, studies on this topic especially focusing on children, are missing to state final recommendations.

## 1. Introduction

Mastocytosis is a disease characterized by growth and build-up of neoplastic mast cells (MCs) in different organ systems. Symptoms are induced by released MC mediators (i.e., skin itching, hives, angioedema, pain in the abdomen and anaphylaxis), or by infiltration of MCs within affected organs, such as skin, bone marrow, gastrointestinal tract, liver and spleen [1,2,3]. The prevalence of mastocytosis has been reported to be 0.9–1.3 per 10,000 inhabitants [4]. Mastocytosis is commonly due to mutations in the KIT receptor, a tyrosine kinase that binds cytokines controlling MC growth. Such mutations result in the activation of the receptor independently from ligand. This leads to proliferation of the MCs [5]. In systemic mastocytosis, the point D816V mutation with substitution of aspartic acid for valine in codon 816 was found in more than 80% of adults but only in one third of children [6]. Mediator release leads to heterogeneous symptoms even if in children the aggressive systemic variant is rare [7]. World Health Organization (WHO) categorized mastocytosis into three different entities: cutaneous mastocytosis, systemic mastocytosis (SM) and mast-cell sarcoma [8]. Cutaneous mastocytosis is the most frequent variant of MC disease in children and it frequently undergoes spontaneous resolution. It is further subcategorized into maculopapular cutaneous mastocytosis (formerly known as urticaria pigmentosa), diffuse cutaneous mastocytosis and localized mastocytoma of the skin [9]. Children are most affected by maculopapular cutaneous mastocytosis, followed by solitary mastocytoma and then by diffuse cutaneous mastocytosis. SM rarely occurs in childhood. Tryptase has a trypsin-like activity, and it represents the main marker of MC disorders. It is stored in the secretory granules of human MCs. It is secreted by MCs, apart from a tiny quantity released from basophils and myeloid precursors. Tryptase levels can differentiate cutaneous mastocytosis from SM. Patients with cutaneous mastocytosis have normal (<11.4 ng/mL), or only slightly augmented serum tryptase concentrations [10] while patients with SM have persistent elevated basal tryptase levels that reflect the extent of body MC involvement [11]. Drug hypersensitivity reactions (DHRs) are a frequent health problem [12]. DHRs are classified as immediate [13], often caused by an IgE-mediated mechanism [14] and nonimmediate, depending on the timeframe between drug intake and occurrence of symptoms. In mastocytosis, quick onset DHRs that are facilitated by MC mediators, are of interest. Immediate DHRs may vary in severity from mild-moderate, such as urticaria-angioedema, rhinitis, conjunctivitis, mild vomiting and diarrhea to bronchospasm, dyspnea, hypotension and anaphylaxis. In adults with MCs disorders drugs are one of the main triggers of HRs after hymenoptera stings. Due to the limited number of children with MCs disease and increased serum tryptase levels, the role of drugs in this age group is less studied. In this review, we critically assessed relevant papers related with immediate DHRs in children with mastocytosis and discuss practical issues of the management.

## 2. Findings in Anaphylactic Reactions

Among DHRs, anaphylaxis is of greater concern since it is life-threatening. The prevalence of both atopy [15,16,17] or IgE-mediated diseases [3] in the general population is similar to that of patients with mastocytosis. Even if the high number of MCs as well as the greater releasability of MCs may increase the risk for anaphylaxis [16,18], the rate of anaphylaxis in children (4%) is much lower than in adults with mastocytosis (49%) [19,20], but it is elevated compared to that in normal children. In children with mastocytosis, anaphylaxis is mainly triggered by drugs and foods [21], even if it is more commonly idiopathic [20,21,22,23,24]. Drugs frequently seem to elicit reactions in nonclonal MC activation syndrome [25]. MC activation and degranulation can be provoked by several stimuli that act through an IgE-independent mechanism or a cross-link of specific IgE-allergen complex on the surface of MCs. A transient elevation of the serum tryptase level by at least 20% over the individual baseline plus 2 ng/mL within 2–4 h after the reaction is considered highly indicative of IgE-mediated MC activation in a patient with signs and symptoms of anaphylaxis [11,26]. Elevated serum tryptase levels are closely correlated to the severity of the reaction [27]. However, many disorders are associated with high basal serum tryptase levels [11], including hereditary alpha tryptasemia that is more frequent in mastocytosis, and it is not associated with an increasing symptoms rate [28]. In mastocytosis, higher serum tryptase concentrations, and widespread skin lesions [29,30], especially during blistering [16,31] increase anaphylaxis risk. Elevated baseline serum tryptase is not associated with augmented frequency of any triggering factor (i.e., hymenoptera venom, drugs and foods) [29]. In patients with SM especially if they suffer from allergic diseases, anaphylaxis is more common than in those with normal serum tryptase level [11]. On the other hand, in cases of systemic symptoms elevated tryptase levels are not always detected. In a retrospective study, tryptase higher than 10 ng/mL was detected in less than 1/4 of 114 children with cutaneous mastocytosis and systemic symptoms [32]. In cases of severe anaphylaxis clonal MC disorders should be excluded. In the differential diagnosis, several factors should be taken into account, in particular basal serum tryptase concentration, skin lesions and clinical manifestations of anaphylaxis. A question is whether epinephrine autoinjectors would be prescribed to children with cutaneous mastocytosis. In a study that enrolled 133 children with maculopapular cutaneous mastocytosis, 69% received an autoinjector prescription but only one patient used it for a reaction to food. This suggests that routine prescription of epinephrine autoinjectors to all children with maculopapular cutaneous mastocytosis should not be recommended [33], but it should be limited to a subgroup of children with extensive skin involvement, high serum tryptase levels, more aggressive cutaneous mastocytosis types and more severe mast cell degranulation symptoms [34].

## 3. Drug Hypersensitivity Reactions in Mastocytosis

Nonsteroidal anti-inflammatory drugs (NSAIDs), antibiotics (beta-lactams, aminoglycosides and streptomycin), phenylephrine, codeine, local and general anesthetics and radiocontrast media, are reported to elicit reactions [15,16,21], even if without confirmation by positive skin tests or challenge tests [35]. Accordingly, MCs activation through MRGPRX2 receptors can be elicited by NSAIDs, succinylcholine, opiates and drugs with tetrahydroisoquinoline (THIQ) motifs including quinolones, atracuronium and rocuronium [36]. Anesthetic drugs, NSAIDs, antibiotics and opioids have been reported to induce fatal anaphylaxis [16,25,37]. Therefore, patients with mastocytosis should be provided with clear recommendations on which drugs should be allowed and which ones should be excluded. Physical stimuli (i.e., heat, cold, quick variations of temperature and rubbing of skin lesions), stress and anxiety can activate MCs [3], misleading the diagnosis in case of concomitant administration of drugs.

### 3.1. General Anesthesia

Children with mastocytosis may undergo sedation or anesthesia and these procedures may induce MC mediator release, through mechanical irritation of the body, stress or administration of medications such as neuromuscular blockers, opioids, hypnotics, analgesics and colloids [38,39,40,41]. These drugs can directly or indirectly activate MCs [42]. From 0.004% to 0.03% of the general population shows perianesthetic anaphilactic or anaphylactoid event [43,44]. In a Spanish retrospective study [45], 501 patients with mastocytosis (including 42 children) underwent 726 anesthetic procedures. The frequency of moderate perioperative symptoms elicited by MC mediator release was low affecting only 4% of children, while three adults (0.4%) and one child (2%) had anaphylaxis. Although peri-operative anaphylaxis in children is generally related to extensive skin involvements (>40% of body surface area) and high concentrations of serum tryptase [16], in this large series no extensive skin involvement, SM or increased serum tryptase levels [45] were shown in the 2 children who developed symptoms. In another retrospective study, 22 children with mastocytosis who received general and local anesthesia and/or sedation, showed only reddening in 9% of cases and gastrointestinal ailments in 18% of cases. Patients did not receive any prophylaxis but continued the chronic antihistamine treatment. An action was not taken in the selection of anesthetics [46]. However, general anesthesia is judged risky for patients with mastocytosis. In case of operations, especially elective, a proper preparation of the patient must be settled down for reducing the risk of peri-operative complications [47]. The anesthetist should be informed about the type of mastocytosis, MC activation grade (e.g., itching infectious diseases or blistering disease) and basal serum tryptase levels. Children similarly to adults, should be prudently anaesthetized when have widespread skin lesions and active blistering skin disease [24]. Patients who have experienced perioperative anaphylaxis [48] are at high risk. Information from previous surgeries should be gained in all patients to offer safe anesthetics. Physical agents (i.e., quick temperature variations or infusion of cold solutions), mechanical factors (i.e., friction or tissue trauma) have a role of paramount importance and avoiding actions should be taken [41]. In general, drugs with the lowest histamine release effect but equally useful should be the first choice. Though it is frequently unpredictable how a patient may respond to the intake of medicines, among the most useful drugs in procedural sedation, propofol, ketamine and midazolam rarely cause MC activation [49,50]. Sevoflurane is a volatile anesthetic that inhibits MC activation, and it is recommended for the maintenance of general anesthesia. It can be given as hypno-inductor especially in children, too [51]. Among the induction agents, that are generally well tolerated, thiopental more commonly induces reactions [41,47]. The available options of analgesic medicines must be well evaluated. Opioids including fentanyl and remifentanil seem sufficiently tolerated for control perioperative pain. It is advisable to slowly administer because they can definitely provoke mediator release from MCs [34,49]. In vitro and in vivo experiments proved that codeine and morphine can potentially induce MC degranulation [52]. Among curaries, the safest for patients with mastocytosis seems to be cis-atracurium. Rocuronium rarely induces degranulation. On the contrary, mivacurium often elicits histamine release, also, in healthy patients and it should not be used [53]. Among the depolarizing neuromuscular blocker, succinylcholine use has been associated to a relatively elevated likelihood of allergic reactions [47]. As preoperative anxiety can play a role in MC degranulation, prevention with benzodiazepines can be given in particular cases. Preoperative drug allergy testing should not be performed in the absence of history of allergic reactions [54]. The risk for perioperative reactions in children with mastocytosis is low and prophylaxis with antiallergic drugs is not mandatory. Prophylaxis with H1 antihistamines, steroids and antileukotrienes should be assessed according to the personal risk. However, maintenance treatment to keep MC stable and limit the results of degranulation should not be suspended. During the perioperative course, the patient with mastocytosis may develop sudden degranulation because of mechanical factors, such as surgery, extreme temperatures or HR to substances and drugs [41]. All patients with perioperative adverse reactions should be investigated with skin testing, serum specific IgE measurement and, when necessary, provocation test to the relevant drugs for ascertaining the diagnosis or selecting a safe alternative. Skin tests are very sensitive for muscle relaxants, less for some antibiotics, barbiturates, benzodiazepines and opioids. False positives can occur with drugs with histamine liberating properties such as curaries (particularly mivacurium, atracurium, cisatracurium and succinylcholine), thiopental, opioids [55]. The intraoperative anaphylactic management [41] in patients with mastocytosis must follow the guidelines for anaphylaxis independently from the underlying disease [56]. Personnel and equipment to cure anaphylactic reactions should be at hand in the surgery room. Moreover, baseline serum tryptase levels should be obtained to improve interpretation of perioperative reactions.

### 3.2. Local Anesthesia

Local anesthesia is commonly well tolerated in all patients including those with mastocytosis, since HR to local anesthetics are exceptional [41,45]. Generally, local anesthetics of the “esters” group should not be used, including procaine, chloroprocaine, tetracaine and benzocaine. Anaphylaxis to local anesthetics of the “amide” group are rare and they can be used with low risk in patients with mastocytosis [53].

### 3.3. Radiocontrast Media

Radiocontrast media (RCM) elicit degranulation of MCs by IgE-mediated mechanism; action on MC membrane because of the high osmolality; nonspecific binding to surface receptors and/or components of the complement system [57]. Investigations on the frequency of anaphylaxis in children with mastocytosis, showed that RCM elicited anaphylaxis in 0 out of 93 children, seven of those with anaphylaxis [15,16,17]. At present, it is not feasible to assess whether RCM is a risk factor of anaphylaxis in patients with mastocytosis because data are lacking [58]. So, there is no evidence that RCM should be avoided. However, as RCM can induce non IgE-mediated, severe reactions in the general population, it seems advisable to prudently premedicate with antihistamines and corticosteroids children with mastocytosis, even its value is undetermined [21].

### 3.4. Antibiotics

Among antibiotics, beta-lactams are the class more frequently inducing HRs in children. When an underlying mastocytosis has to be suspected it is not clear. Most of the published studies suggest to investigate basal tryptase levels in cases of drug reactions with a severe presentation [37]. So far, limited evidence exists on the tolerance of antibiotics in children with mastocytosis. Further studies with provocation challenges [59] are necessary to elucidate this issue. A trial studied 133 children with cutaneous mastocytosis. Reactions to medications (6 to beta-lactams, 3 to acetaminophen and 2 to measles, mumps, and rubella (MMR) vaccine) were recorded in 12 patients [60]. An 8 years old patient with increased serum basal tryptase level was reported to develop anaphylaxis with loss of consciousness after ceftriaxone use [23]. In a 13-year-old girl [61], the occurrence of anaphylaxis during general anesthesia was associated to mastocytosis. Midazolam, fentanyl, lidocaine and propofol were used to anaesthetize the girl during an orthopedic surgery. She had wheezing, generalized rash and hypotension two minutes after the following administration of cefazolin. An allergy work-up was not performed. Castells observed a 4-month-old child with generalized hemorrhagic blistering after vancomycin use. It was administered to treat a febrile disease [62]. Highly elevated tryptase level of 180 ng/mL were found. It is already recognized that vancomycin is considered a trigger of MC release.

### 3.5. Nonsteroidal Anti-Inflammatory Drugs

NSAIDs besides beta-lactams are the most common causes of drug induced anaphylaxis both in adults and children [63]. Additionally, NSAIDs act as cofactors in patients with food associated exercise-dependent anaphylaxis [64] or in patients with food allergy [65] by augmenting gut permeability to food allergens [66]. Elevated basal serum tryptase, and widespread skin lesions were risk factors for severe symptoms in 111 children with cutaneous mastocytosis. However, only 4 children had immediate HRs to drugs. A child had only itching, a child had urticaria without angioedema and two children had urticaria and angioedema. The offending medicines were dipyrone and diclofenac, ibuprofen and a polivitaminic complex [67].

In a Spanish series of 96 children with mastocytosis only 2 (2%) of them had HRs to aspirin or other NSAIDs (pyrazolone, diclofenac, ibuprofen and naproxen) [68]. Symptoms described were anaphylaxis, followed by urticaria/angioedema, flushing and asthma.

In children with mastocytosis the risk of severe systemic reactions after NSAIDs intake seems to be very low and in general lower than in adults. Anyway, studies on this topic especially focusing on children, are missing to state final recommendations.

As for adults also children who tolerated NSAIDs before the diagnosis of SM, continue to safely receive these medications and elimination should not be suggested.

### 3.6. Other Drugs

Monoclonal antibodies have been given in a relatively small number of patients with mastocytosis without any report of anaphylaxis in the literature. Omalizumab has been effectively administered to avoid HRs in patients with allergy to hymenoptera venom, venom immunotherapy HR or idiopathic anaphylaxis. Regarding opioids, published data are scarce in adults and no data are available in children. Commonly in patients with mastocytosis, it is advisable to avoid histamine releasing drugs such as morphine or buprenorphine [69]. In particular, in vitro studies have demonstrated that among this class of drugs, opioids differ in their capacity to provoke histamine release by MCs, for example fentanyl dose not induce histamine release and it should be preferred over morphine for pain relief [21]. No data on chemotherapy or other drugs are available in the pediatric population [70].

## 4. Vaccines for Infectious Diseases

Vaccines for infectious diseases may trigger MC mediator release in patients with mastocytosis, but the rate of such HRs is unknown. In small population samples, vaccines have been reported to trigger adverse reactions in 6–13% of children with mastocytosis [71,72,73]. According to National Institute Health (NIH) retrospective data, the incidence of adverse reactions to vaccination was 1.8% and the prevalence 8% in 75 children with mastocytosis [1]. However, a working group report found an adverse reaction rate of 0.6% in 634 children with mastocytosis showed. This was similar to that in the healthy population [22]. The most common reactions were large local cutaneous reactions. Furthermore, anaphylaxis has not been clearly associated with vaccine administration, only one case of anaphylaxis was related to vaccination (Table 1). HRs occurred with both live and attenuated vaccines and did not habitually return with boosters. In a child who had a HR, a modified schedule may be effective for recognizing the offending antigen [74]. A generalized bullous reaction provoked by the first administration of a multivalent vaccine was reported in a 5-month-old infant with diffuse cutaneous mastocytosis [75]. Moreover, in 2 children [76,77], a mastocytoma developed at the place where it was inoculated hepatitis B vaccine. Some vaccine ingredients, including dextran, gelatin, and polymyxin B, may be responsible for HRs, but the mechanisms inducing release of MC mediators is unclear [78]. Probably vaccines components may act as superantigen by triggering MC degranulation after an unspecific binding to human IgE. Most of the studies published so far showed no further reaction with another dose of the same vaccine. This fact could be possibly explained with the hypothesis that a development of IgM or IgG which neutralized the superantigens before they interact with IgE, may occur. Some authors proved the efficacy of premedication after a reaction to vaccination in children with mastocytosis. Recently a review has been published on the issue [69], but no conclusion has been reached on the utility of premedication and on the schemes of premedication since controlled studies are lacking. Regarding mRNA COVID-19 vaccines there is no evidence that they may elicit adverse reactions in children with mastocytosis. However, it has been cautiously suggested in adults to consider a preventive treatment with sedating or non-sedating H1 antihistamine given 30–60 min before vaccination, and, also, with H2-antihistamines or montelukast and post-vaccination observation [79]. Moreover, regarding the possibility of splitting vaccines, ministerial Italian recommendations [72] and experts in the field discourage the simultaneous administration of multiple vaccines [73], and making use of polyvalent vaccines.

Overall, counseling for parents should highlight that there is a low likelihood of life-threatening HRs to vaccines in children with mastocytosis. Therefore, they should receive jabs according to the routine schedule except if HRs previously occurred or the child is allergic to a vaccine ingredient.

## 5. Discussion

In conclusion, although the chance of anaphylaxis in patients with mastocytosis is higher than in the general population, it is lower than most physicians predict. Given that anaphylaxis in children with mastocytosis is more commonly idiopathic, the importance of drugs as cause of anaphylaxis is overemphasized, and physical or mechanical factors are at least equally significant in provoking MC degranulation. Drugs, level of basal tryptase and high burden of disease do not increase the risk. In children who did not experience previous HR, it is not necessary to withhold any medications. Regarding surgery, routinary pre-operative drug testing are not recommended. Anyway, precautionary measures such as a meticulous history, the substitution of offending medicines, and a careful action plan to control HRs during anesthetics and RCM administration are advisable. In children with mastocytosis, the efficacy of premedication for RCM and general anesthetics has not been properly assessed in high quality studies so it should be discussed with patients. At the same time, chronic antimediator drugs should be continued once started. In children with mastocytosis other drugs including antibiotics, NSAIDs and opioids can potentially induce anaphylaxis. So far there are few studies on children without a systematic evaluation, so no firm conclusions can be reached. Among drugs, NSAIDs are the leading trigger of anaphylaxis or may exacerbate other atopic diseases. Anyway only 0–2% of children with mastocytosis, may develop HRs to NSAIDs. Patients who tolerated NSAIDs before occurrence of SM can go on using them. The evidence is lacking to avoid NSAIDs and for pre-test evaluation in children with mastocytosis. Last but not at least, mastocytosis does not contraindicate vaccination [80], also taking into account that children with mastocytosis have a slightly increased risk of reaction in comparison with the general population [70]. Reactions are frequently mild with exceedingly rare bullous eruptions or more severe cases of MC degranulation. Although the proofs are weak, children with extensive skin disease, (i.e., diffuse cutaneous mastocytosis), may be at higher risk of reaction to vaccines especially to hexavalent ones and to the first injection. For that reason, in patients with diffuse cutaneous mastocytosis and extensive skin involvement the first injection should be performed in a controlled setting and single-vaccine regimens can be considered. Children should be observed for 2 h following vaccination, and patients or their parents should be educated on how ask for additional medical care.

## 6. Conclusions

In conclusion when dealing with mastocytosis a multidisciplinary approach involving pediatricians, allergists, immunologists, dermatologists, anesthetists, radiologists and dental doctors based on the setting and individual case, is of paramount importance to manage safely the patient and provide correct information regarding drug intake or vaccination.

## Figures and Tables

**Table 1:** Reactions to vaccination in children with mastocytosis (modified from 64).

Authors	Patients with Reactions/Total Sample	Variant of Mastocytosis/Age of Diagnosis (Years)	Eliciting Vaccine (Number of Doses Received)	Reaction/Time Interval	Subsequent Vaccines/ Premedication
Zanoni et al. [66]	7/102 (35 children)	Mastocytoma/0.5 Mastocytoma/0.4 Mastocytoma/2 Mastocytoma/0.6 MPCM/0.2 MPCM/0.5 DCM/0.4	Hexavalent (1) Hexavalent (2) MenC (1) Hexavalent (3) PCV (2), MenC (1) HPV (1) Hexavalent (1,2), PCV (1, 2)	Urticaria and angioedema/20 min Local and facial flushing/20 min Fever and gastrointestinal clinical manifestations/24 h Injection site reaction and fever/8 h Fever/8 h Hives on arm and nasal obstruction/12 h Hives and itch on trunk and febrile convulsions/12 h	DTaP, IPV, HB, Hib, MMRV/not available Hexavalent/not available PCV/not available
Parente et al. [69]	4/72 children	MPCM/0.3 Mastocytoma/2 Mastocytoma/4 DCM/0.1	Hexavalent (1) Hexavalent (1) Hexavalent (1) Hexavalent (1)	Bullous skin reaction/6–12 h Diffuse urticaria/1–4 h Diffuse urticaria/1–4 h Bullous skin reaction and mild bronchospasm/6–12 h	Other mandatory vaccines/oral antihistamines Other mandatory vaccines Other mandatory vaccines Other mandatory vaccines/oral antihistamines
Bankova et al. [70]	1 child	DCM	DTaP IPV HiB Rotavirus	Confluent blisters on the back, abdomen, and upper arms/a day later	Unknown/oral and topical sodium cromolyn
Johansen et al. [71]	1 child3/35 children	MPCM MPCM MPCM	DTaP IPV HiB Rotavirus DTaP All vaccines	Skin flushing, itch, blisters, gastrointestinal clinical manifestations/hours Skin flushing and pruritus/minutes Fever/hours	
Sarcina et al. [64]	1 child	MPCM	inactivated tetravalent influenza vaccine (2)	diffuse bullous cutaneous reaction/24 h	PCV13; V vaccines oral antihistamines and betamethasone (background therapy with ketotifen)

DCM, diffuse cutaneous mastocytosis; Hexavalent, diphtheria–tetanus toxoid–acellular pertussis (DTaP)–hepatitis B (HB)–inactivated polio vaccine (IPV)–Haemophilus influenzae B vaccine (HiB); h, hours; HPV, human papilloma virus vaccine; IPV, inactivated polio vaccine; min, minutes; MPCM, maculopapular cutaneous mastocytosis; Men C, meningococcal C vaccine; MMRV, measles, mumps, rubella, varicella vaccine; PCV, pneumococcal vaccine.

## References

[1] Hussain S.H. (2020). Pediatric mastocytosis. Curr. Opin. Pediatr..

[2] Theoharides T.C., Valent P., Akin C. (2015). Mast cells, mastocytosis, and related disorders. N. Engl. J. Med..

[3] Escribano L., Orfao A., Castells M.C. (2011). Anaphylaxis in mastocytosis. Anaphylaxis and Hypersensitivity Reactions.

[4] Brockow K. (2014). Epidemiology, prognosis, and risk factors in mastocytosis. Immunol. Allergy Clin. N. Am..

[5] Nagata H., Worobec A.S., Oh C.K., Howdhury B.A., Tannenbaum S., Suzuki Y., Metcalfe D.D. (1995). Identification of a point mutation in the catalytic domain of the protooncogene c-kit in peripheral blood mononuclear cells of patients who have mastocytosis with an associated hematologic disorder. Proc. Natl. Acad. Sci. USA.

[6] Metcalfe D.D. (2008). Mast cells and mastocytosis. Blood.

[7] Hoermann G., Gleixner K.V., Dinu G.E., Kundi M., Greiner G., Wimazal F., Hadzijusufovic E., Mitterbauer G., Mannhalter C., Valent P. (2014). The KIT D816V allele burden predicts survival in patients with mastocytosis and correlates with the WHO type of the disease. Allergy.

[8] Valent P., Akin C., Dean D. (2017). Mastocytosis: 2016 updated WHO classification and novel emerging treatment concepts. Blood.

[9] Hoeger P. (2019). Pediatric mastocytosis. Harper’s Textbook of Pediatric Dermatology.

[10] Bonadonna P., Lombardo C., Zanotti R. (2014). Mastocytosis and allergic diseases. J. Investig. Allergol. Clin. Immunol..

[11] Valent P., Bonadonna P., Hartmann K., Broesby-Olsen S., Brockow K., Butterfield J.H., Triggiani M., Lyons J.J., Oude Elberink J.N.G., Arock M. (2019). Why the 20% + 2 tryptase formula is a diagnostic gold standard for severe systemic mast cell activation and mast cell activation syndrome. Int. Arch. Allergy Immunol..

[12] Gomes E., Cardoso M.F., Praca F., Gomes L., Marino E., Demoly P. (2004). Self-reported drug allergy in a general adult Portuguese population. Clin. Exp. Allergy.

[13] Blanca M., Romano A., Torres M.J., Fernandez J., Mayorga C., Rodriguez J., Demoly P., Bousquet P.J., Merk H.F., Sanz M.L. (2009). Update on the evaluation of hypersensitivity reactions to betalactams. Allergy.

[14] Bircher A.J., Scherer Hofmeier K. (2012). Drug hypersensitivity reactions: Inconsistency in the use of the classification of immediate and nonimmediate reactions. J. Allergy Clin. Immunol..

[15] González de Olano D., de la Hoz Caballer B., Núñez López R., Sánchez M., Cuevas Agustín M., Diéguez M.C., Alvarez Twose I., Castells M.C., Escribano Mora L. (2007). Prevalence of allergy and anaphylactic symptoms in 210 adult and pediatric patients with mastocytosis in Spain: A study of the Spanish network on mastocytosis (REMA). Clin. Exp. Allergy.

[16] Brockow K., Jofer C., Behrendt H., Ring J. (2008). Anaphylaxis in patients with mastocytosis: A study on history, clinical features and risk factors in 120 patients. Allergy.

[17] Gulen T., Hagglund H., Dahlen B., Nilsson G. (2014). High prevalence of anaphylaxis in patients with systemic mastocytosis: A singlecentre experience. Clin. Exp. Allergy.

[18] Schuch A., Brockow K. (2017). Mastocytosis and anaphylaxis. Immunol. Allergy Clin. N. Am..

[19] Gurnee E., Phung T., Guo E., Fong A., Tollefson M., Nguyen H., Brandling-Bennett H.A., Moriarty N., Paller A.S., Huynh T. (2020). Neurocognitive dysfunction and anaphyklaxis in pediatric maculopapular cutaneous mastocytosis. J. Allergy Clin. Immunol..

[20] Brockow K., Plata-Nazar K., Lange M., Nedoszytko B., Niedoszytko M., Valent P. (2021). Mediator-related symptoms and anaphylaxis in children with mastocytosis. Int. J. Mol. Sci..

[21] Bonadonna P., Pagani M., Aberer W., Bilò M.B., Brockow K., Oude Elberink H., Garvey L., Mosbech H., Romano A., Zanotti R. (2015). Drug hypersensitivity in clonal mast cell disorders: ENDA/EAACI position paper. Allergy.

[22] Carter M.C., Clayton S.T., Komarow H.D., Brittain E.H., Scott L.M., Cantave D., Gaskins D.M., Maric I., Metcalfe D.D. (2015). Assessment of clinical findings, tryptase levels, and bone marrow histopathology in the management of pediatric mastocytosis. J. Allergy Clin. Immunol..

[23] Bonadonna P., Zanotti R., Pagani M., Caruso B., Perbellini O., Colarossi S., Olivieri E., Dama A., Schiappoli M., Senna G. (2009). How much specific is the association between Hymenoptera venom allergy and mastocytosis?. Allergy.

[24] Brockow K., Bonadonna P. (2012). Drug allergy in mast cell disease. Curr. Opin. Allergy Clin. Immunol..

[25] Alvarez-Twose I., González de Olano D., Sánchez-Muñoz L., Matito A., Esteban-López M.I., Vega A., Mateo M.B., Alonso Díaz de Durana M.D., de la Hoz B., Del Pozo Gil M.D. (2010). Clinical, biological, and molecular characteristics of clonal mast cell disorders presenting with systemic mast cell activation symptoms. J. Allergy Clin. Immunol..

[26] Schwartz L.B., Metcalfe D.D., Miller J.S., Earl H., Sullivan T. (1987). Tryptase levels as an indicator of mast cell activation in systemic anaphylaxis and mastocytosis. N. Engl. J. Med..

[27] Van der Linden P.W., Hack C.E., Poortman J., Vivie-Kipp Y.C., Struyvenberg A., van der Zwan J.K. (1992). Insect sting challenge in 138 patients: Relation between clinical severity of anaphylaxis and mast cell activation. J. Allergy Clin. Immunol..

[28] Chollet M.B., Akin C. (2022). Hereditary alpha tryptasemia is not associated with specific clinical phenotypes. J. Allergy Clin. Immunol..

[29] Fellinger C., Hemmer W., Worhl S., Sesztak-Greinecker G., Jarisch R., Wantke F. (2014). Clinic characteristics and risk profile of patients with elevated basal serum tryptase. Allergol. Immunopathol..

[30] Alvarez-Twose I., Zanotti R., Gonzalez-de-Olano D., Bonadonna P., Vega A., Matito A., Sánchez-Muñoz L., Morgado J.M., Perbellini O., García-Montero A. (2014). Nonaggressive systemic Mastocytosis (SM) without skin lesions associated with insect-induced anaphylaxis shows unique features versus other indolent SM. J. Allergy Clin. Immunol..

[31] Brockow K., Ring J., Alvarez-Twose I., Orfao A., Escribano L. (2012). Extensive blistering is a predictor for severe complications in children with mastocytosis. Allergy.

[32] Seth N., Chinen J., Buheis G., Buheis M.G., Chan A.J., Hunt R.D., Tuano K.T. (2018). Serum tryptase levels in 114 pediatric patients with cutaneous mastocytosis. J. Allergy Clin. Immunol..

[33] Cardona V., Ansotegui I.J., Ebisawa M., El-Gamal Y., Fernandez Rivas M., Fineman S., Geller M., Gonzalez-Estrada A., Greenberger P.A., Sanchez Borges M. (2020). World allergy organization anaphylaxis guidance 2020. World Allergy Organ J..

[34] Simons F.E. (2009). Anaphylaxis: Recent advances in assessment and treatment. J. Allergy Clin. Immunol..

[35] Bonadonna P., Lombardo C. (2014). Drug allergy in mastocytosis. Immunol. Allergy Clin. N. Am..

[36] McNeil B.D., Pundir P., Meeker S., Han L., Undem B.J., Kulka M., Dong X. (2015). Identification of a mast-cell-specific receptor crucial for pseudo-allergic drug reactions. Nature.

[37] Bonadonna P., Perbellini O., Passalacqua G., Caruso B., Colarossi S., Dal Fior D., Castellani L., Bonetto C., Frattini F., Dama A. (2009). Clonal mast cell disorders in patients with systemic reactions to Hymenoptera stings and increased serum tryptase levels. J. Allergy Clin. Immunol..

[38] Marone G., Stellato C. (1992). Activation of human mast cells and basophils by general anaesthetic drugs. Monogr. Allergy.

[39] Stellato C., de Paulis A., Cirillo R., Mastronardi P., Mazzarella B., Marone G. (1991). Heterogeneity of human mast cells and basophils in response to muscle relaxants. Anesthesiology.

[40] Stellato C., Marone G. (1995). Mast cells and basophils in adverse reactions to drugs used during general anesthesia. Chem. Immunol..

[41] Dewachter P., Castells M.C., Hepner D.L., Mouton-Faivre C. (2014). Perioperative management of patients with mastocytosis. Anesthesiology.

[42] Greenblatt E.P., Chen L. (1990). Urticaria pigmentosa: An anesthetic challenge. J. Clin. Anesth..

[43] Currie M., Webb R.K., Williamson J.A., Russell W.J., Mackay P. (1993). The Australian Incident Monitoring Study: Clinical anaphylaxis—An analysis of 2, 000 incident reports. Anaesth. Intensive Care.

[44] Kroigaard M., Garvey L.H., Gillberg L., Johansson S.G., Mosbech H., Florvaag E., Harboe T., Eriksson L.I., Dahlgren G., Seeman-Lodding H. (2007). Scandinavian clinical practice guidelines on the diagnosis, management and follow-up of anaphylaxis during anaesthesia. Acta Anaesthesiol. Scand..

[45] Matito A., Morgado J.M., Sánchez-López P., Álvarez-Twose I., Sánchez-Muñoz L., Orfao A., Escribano L. (2015). Management of anesthesia in adult and pediatric mastocytosis: A study of the Spanish Network on Mastocytosis (REMA) based on 726 anesthetic procedures. Int. Arch. Allergy Immunol..

[46] Carter M.C., Uzzaman A., Scott L.M., Metcalfe D.D., Quezado Z. (2008). Pediatric mastocytosis: Routine anesthetic management for a complex disease. Anesth. Analg..

[47] Mertes P.M. (2011). Reducing the risk of anaphylaxis duringanesthesia: 2011 Updated guidelines for clinical practice. J. Investig. Allergol. Clin. Immunol..

[48] Caimmi S., Caimmi D., Bernardini R., Caffarelli C., Crisafulli G., Pingitore G., Marseglia G.L. (2011). Perioperative anaphylaxis: Epidemiology. Int. J. Immunopathol. Pharmacol..

[49] Borgeat A., Ruetsch Y.A. (1998). Anesthesia in a patient with malignant systemic mastocytosis using a total intravenous anesthestic technique. Anesth. Analg..

[50] Ulbrich F., Engelstädter H., Wittau N., Steinmann D. (2013). Anaesthetic management of emergency caesarean section in a parturient with systemic mastocytosis. Int. J. Obstet. Anesth..

[51] Auvray L. (2001). Mastocytose: Anesthésie générale par rémifentanil et sévoflurane. Ann. Fr. Anesth. Reanim..

[52] Sheen C.H., Schleimer R.P., Kulka M. (2007). Codeine induces human mast cell chemokine and cytokine production: Involvement of G-protein activation. Allergy.

[53] Bonadonna P., Zanotti R., Bisoffi Varani A., Pagani M. (2017). Mast cell diseases and drug hypersensitivity reactions. Curr. Treat Options. Allergy.

[54] Pardanani A. (2013). How I treat patients with indolent and smoldering mastocytosis (rare conditions but difficult to manage). Blood.

[55] Stepanovic B., Sommerfield D., Lucas M., von Ungern-Sternberg B.S. (2019). An update on allergy and anaphylaxis in pediatric anesthesia pediatric anesthesia. Paediatr. Anaesth..

[56] Caffarelli C., Ricò S., Rinaldi L., Povesi Dascola C., Terzi C., Bernasconi S. (2012). Blood pressure monitoring in children undergoing food challenge: Association with anaphylaxis. Ann. Allergy Asthma Immunol..

[57] Palmiere C., Reggiani Bonetti L. (2014). Risk factors in fatal cases of anaphylaxis due to contrast media: A forensic evaluation. Int. Arch. Allergy Immunol..

[58] Bonadonna P., Bonifacio M., Zanotti R. (2016). Mast cell disorders in drug hypersensitivity. Curr. Pharm. Des..

[59] Caffarelli C., Franceschini F., Caimmi D., Mori F., Diaferio L., Di Mauro D., Mastrorilli C., Arasi S., Barni S., Bottau P. (2018). SIAIP position paper: Provocation challenge to antibiotics and non-steroidal anti-inflammatory drugs in children. Ital. J. Pediatr..

[60] Schena D., Galvan A., Tessari G., Girolomoni G. (2016). Clinical features and course of cutaneous mastocytosis in 133 children. Br. J. Dermatol..

[61] Goldfinger M.M., Sandadi J. (2010). Undiagnosed systemic mastocytosis in a teenager revealed during general anesthesia. Paediatr. Anaesth..

[62] Molderings G.J., Brettner S., Homann J., Afrin L.B. (2011). Mast cell activation disease: A concise practical guide for diagnostic workup and therapeutic options. J. Hematol. Oncol..

[63] Aun M.V., Blanca M., Garro L.S., Ribeiro M.R., Kalil J., Motta A.A., Castells M., Giavina-Bianchi P. (2014). Nonsteroidal anti-inflammatory drugs are major causes of drug-induced anaphylaxis. J. Allergy Clin. Immunol. Pract..

[64] Ricci G., Andreozzi L., Cipriani F., Giannetti A., Gallucci M., Caffarelli C. (2019). Wheat allergy in children: A comprehensive update. Medicina.

[65] Pfeffer I., Fischer J., Biedermann T. (2011). Acetylsalicylic acid dependent anaphylaxis to carrots in a patient with mastocytosis. J. Dtsch. Dermatol. Ges..

[66] Morita E., Kunie K., Matsuo H. (2007). Food-dependent exercise-induced anaphylaxis. J. Dermatol. Sci..

[67] Alvarez-Twose I., Vano-Galvan S., Sanchez-Munoz L., Morgado J.M., Matito A., Torrelo A., Jaén P., Schwartz L.B., Orfao A., Escribano L. (2012). Increased serum baseline tryptase levels and extensive skin involvement are predictors for the severity of mast cell activation episodes in children with mastocytosis. Allergy.

[68] Seitz C.S., Brockow K., Hain J., Trautmann A. (2014). Non-steroidal anti-inflammatory drug hypersensitivity: Association with elevated basal serum tryptase?. Allergy Asthma Clin. Immunol..

[69] Stellato C., Cirillo R., de Paulis A., Casolaro V., Patella V., Mastronardi P., Mazzarella B., Marone G. (1992). Human basophil/mast cell releasability. Anesthesiology.

[70] Lange M., Hartmann K., Carter M.C., Siebenhaar F., Alvarez-Twose I., Torrado I., Brockow K., Renke J., Irga-Jaworska N., Plata-Nazar K. (2021). Molecular Background, Clinical Features and Management of Pediatric Mastocytosis: Status 2021. Int J. Mol. Sci..

[71] Sarcina D., Giovannini M., Oranges T., Barni S., Pedaci F.A., Liccioli G., Canessa C., Sarti L., Lodi L., Filippeschi C. (2021). Case report and review of the literature: Bullous skin eruption after the booster-dose of Influenza Vaccine in a pediatric patient with polymorphic maculopapular cutaneous mastocytosis. Front. Immun..

[72] Istituto Superiore di Sanità Guida Alle Controindicazioni Alle Vaccinazioni. http://www.salute.gov.it/portale/documentazione/p6_2_2_1.jsp?.

[73] Zanoni G., Zanotti R., Schena D., Sabbadini C., Opri R., Bonadonna P. (2017). Vaccination management in children and adults with mastocytosis. Clin. Exp. Allergy.

[74] Clark A., Metcalfe D., Scott L., Carter M. (2015). Adverse vaccine reactions in children with pediatric mastocytosis. J. Allergy Clin. Immunol..

[75] Bankova L.G., Walter J.E., Iyengar S.R., Lorenzo M.E., Hornick J.L., Castells M.C. (2013). Generalized bullous eruption after routine vaccination in a child with diffuse cutaneous mastocytosis. J. Allergy Clin. Immunol. Pract..

[76] Koh M.J., Chong W.S. (2008). Red plaque after hepatitis B vaccination. Pediatr. Dermatol..

[77] Poulton J.K., Kauffman C.L., Lutz L.L., Sina B. (1999). Solitary mastocytoma arising at a hepatitis B vaccination site. Cutis.

[78] Franceschini F., Bottau P., Caimmi S., Cardinale F., Crisafulli G., Liotti L., Pellegrini G., Peroni D., Saretta F., Mastrorilli C. (2018). Evaluating children with suspected allergic reactions to vaccines for infectious diseases. Allergy Asthma. Proc..

[79] Bonadonna P., Brockow K., Niedoszytko M., Elberink H.O., Akin C., Nedoszytko B., Butterfield J.H., Alvarez-Twose I., Sotlar K., Schwaab J. (2021). COVID-19 Vaccination in mastocytosis: Recommendations of the European Competence Network on Mastocytosis (ECNM) and American Initiative in Mast Cell Diseases (AIM). J. Allergy Clin. Immunol. Pract..

[80] Johasen M.L., Lawley L.P. (2021). Assessing vaccination reactions in pediatric patients with maculopapular cutaneous mastocytosis. Pediatr. Dermatol..

